# Real‐world effectiveness and safety of abrocitinib in 12 Japanese patients with atopic dermatitis and transcriptome analysis with peripheral blood

**DOI:** 10.1111/1346-8138.17173

**Published:** 2024-03-03

**Authors:** Akihiko Uchiyama, Keiji Kosaka, Mai Ishikawa, Yuta Inoue, Sei‐ichiro Motegi

**Affiliations:** ^1^ Department of Dermatology Gunma University Graduate School of Medicine Maebashi Gunma Japan

**Keywords:** abrocitinib, atopic dermatitis, Japanese, real‐world data, RNA‐seq

## Abstract

Atopic dermatitis (AD) is a common chronic inflammatory skin disease characterized by recurrent, pruritic, and localized eczema. Various types of new drugs have been recently investigated for treating AD. The efficacy and safety of abrocitinib in treating AD has been reported in clinical trials, but the real‐world data from Japan has not been reported. Herein, we analyzed 12 Japanese patients with AD treated with 100 mg of abrocitinib using our real‐world data. We also performed transcriptome analysis with peripheral blood to investigate the effects of abrocitinib on cytokine expressions and inflammatory pathways in AD from three patients. This study included patients with moderate to severe AD treated with abrocitinib at Gunma University Hospital, Japan. All patients were systemic treatment‐naïve. All patients received a 100‐mg dose of abrocitinib daily, and used strong or very strong topical steroids and moisturizers. The Eczema Area and Severity Index (EASI) response analysis revealed that after 4 weeks, 25% (three of 12) of the cases reached a 75% reduction in the EASI score (EASI‐75) and a 90% reduction in the EASI score (EASI‐90). After 12 weeks, 83.3.% (10 of 12), 41.6% (five of 12), and 16.7% (two of 12) of the patients reached EASI‐50, a 75% reduction in the EASI score (EASI‐75), and EASI‐90. Peak Pruritus Numerical Rating Scale was achieved in nine patients (75%) at week 12. The most frequent adverse reaction was acne (six cases [50%]). Gene Ontology pathway analysis using Differentially expressed genes from RNA sequencing analysis revealed attenuation of defense responses to biotic stimulus, virus, and cytokines. Th2 cytokine expression was not suppressed, but several chemokines, especially CXCL1, were suppressed by abrocitinib treatment. Our results indicate abrocitinib as a fast‐acting and highly antipruritic agent that is effective for moderate skin eruptions.

## 1 INTRODUCTION

Atopic dermatitis (AD) is a common chronic inflammatory skin disease characterized by recurrent, pruritic, and localized eczema.^1^ Various types of new drugs have been recently investigated for treating AD. In September 2021, abrocitinib, an oral selective Janus kinase (JAK) 1 inhibitor, was approved for moderate to severe AD in patients older than 12 years in Japan. Clinical trials and meta‐analyses reported the efficacy and safety of abrocitinib in treating AD.^2^, ^3^, ^4^, ^5^, ^6^ However, no report has revealed its efficacy and safety using real‐world evidence from Japan. Herein, we analyzed 12 Japanese patients with AD treated with a 100‐mg dose of abrocitinib using our real‐world data. We also performed transcriptome analysis with peripheral blood to investigate the effects of abrocitinib on cytokine expressions and inflammatory pathways in AD from three patients.

## 2 METHODS

This study included patients with moderate to severe AD treated with abrocitinib from September 2021 to November 2023 at Gunma University Hospital, Japan. All patients fulfilled the diagnosis criteria according to the Japanese guidelines for AD.^7^ All patients were systemic treatment‐naïve. Twelve patients completed the 12‐week treatment without any interruption at our hospital. All patients received a 100‐mg dose of abrocitinib daily and used strong or very strong topical steroids and moisturizers. The institutional review board of Gunma University (HS2022‐169) approved this retrospective cohort study, which was conducted under the principles of the Declaration of Helsinki. Demographic and disease characteristics of AD (Investigator's Global Assessment [IGA], affected body surface area, Eczema Area and Severity Index [EASI], total and head/neck, Peak Pruritus Numerical Rating Scale [PP‐NRS], and Patient‐Oriented Eczema Measure [POEM]); serum markers; and adverse events. All these characteristics were assessed at the following time points: baseline, 4 weeks, and 12 weeks. Four weeks before and after abrocitinib treatment, peripheral blood mononuclear cells were obtained from patients number 1, 2, and 4. The RNeasy Mini kit (QIAGEN) was used to collect total RNA from peripheral blood mononuclear cells, following the manufacturer's protocol. NextSeq 500 System (Illumina Inc.) with a NextSeq 500 High Output v2.5 Kit (Illumina) was used for RNA sequencing (RNA‐seq) and Ingenuity Pathway Analysis (QIAGEN) was used for pathway analysis, as previously described.^8^

## 3 RESULTS

**FIGURE 1:**
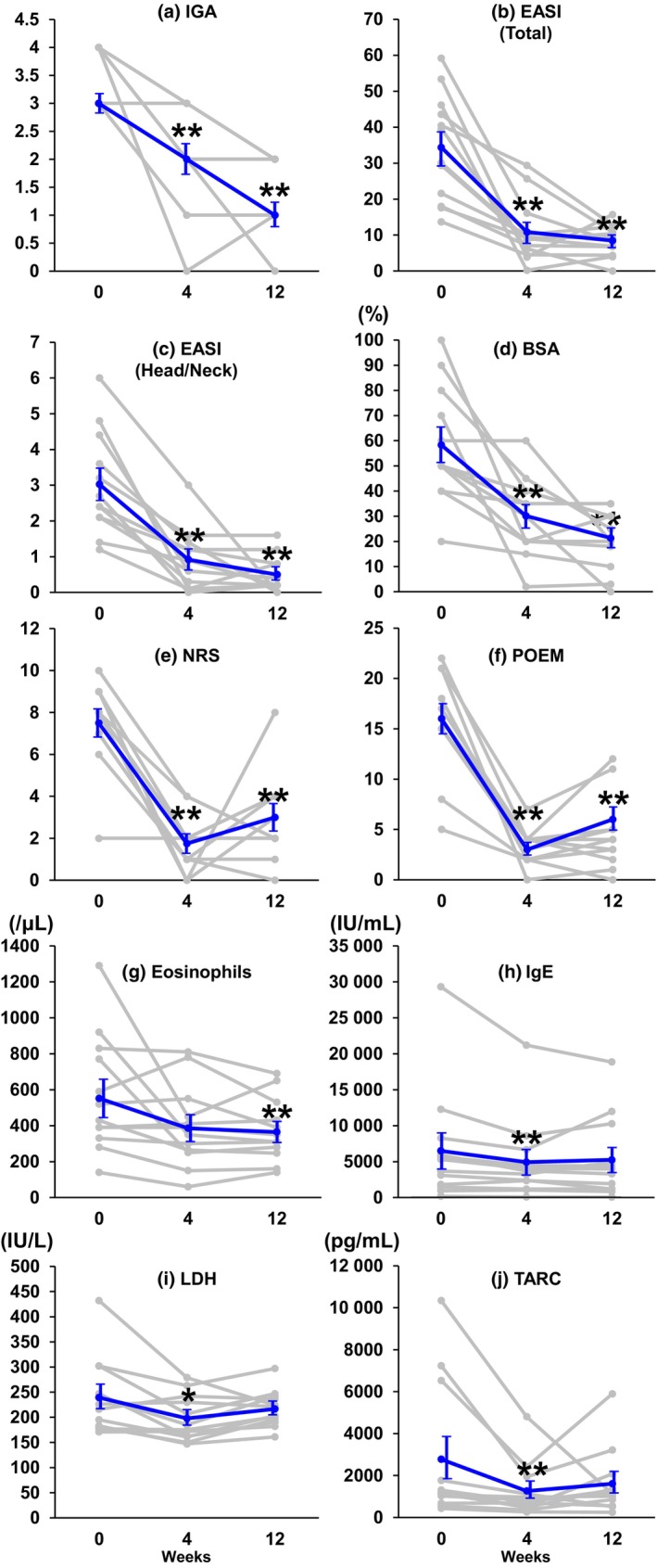
Change in efficacy outcomes and serum markers after 4 and 12 weeks. The line chart shows individual data (gray line) and average (blue) changes in each score at baseline, week 4, and week 12 after treatment with abrocitinib (*n* = 12). (a) Investigator's Global Assessment (IGA). (b, c) Eczema Area and Severity Index (EASI) (b) total and (c) head/neck. (d) Affected body surface area (BSA). (e) Peak Pruritus Numerical Rating Scale (NRS). (f) Patient‐Oriented Eczema Measure (POEM). (g) Numbers of eosinophils in peripheral blood. (h) Serum IgE. (i) Serum thymus and activation‐regulated chemokine (TARC). (j) Serum lactate dehydrogenase (LDH). Statistical analyses were performed using Prism 10 software (GraphPad Software), Wilcoxon matched‐pairs signed rank test, and Pearson correlation coefficient. Values represent mean ± SEM. *p < 0.05, **p < 0.01.

The Table 1 and Table S1 summarize the baseline demographics and clinical characteristics of the patients. This study analyzed five adolescent and seven adult patients, including eight males and four females, with a mean age ± SD of 29.8 ± 18.7 years. Six (50%) cases were classified as moderate (IGA = 3) and six cases (50%) were identified as severe (IGA = 4). Clinical and laboratory data at baseline and changes observed at both time points of 4 and 12 weeks after initiating abrocitinib demonstrated a significant decrease in IGA, body surface area, and EASI (total and head/neck) scores (Figure 1 and Figure S1). The EASI response analysis revealed that after 4 weeks, 25% (three of 12) of the cases reached a 75% reduction in the EASI score (EASI‐75) and a 90% reduction in the EASI score (EASI‐90). After 12 weeks, 83.3% (10 of 12), 41.6% (five of 12), and 16.7% (two of 12) of the patients reached a 50% reduction in the EASI score (EASI‐50), EASI‐75, and EASI‐90. Patient‐reported symptoms (POEM/PP‐NRS) significantly decreased at 4 and 12 weeks compared with the baseline condition. However, they were slightly worse at 12 weeks than at 4 weeks. The eosinophil count in the peripheral blood was significantly decreased at 12 weeks. Serum lactate dehydrogenase, thymus and activation‐regulated chemokine, and serum IgE were significantly decreased at 4 weeks but not at 12 weeks. Adverse effects were observed as described: acne (six [50%] cases), elevated creatine kinase levels (two [16.7%] cases), headache (one [8.3%] case), nausea (one [8.3%] case), and diarrhea (one [8.3%] case). All patients continued treatment despite these adverse effects.

**TABLE 1:** Patient demographics and baseline disease characteristics.

Characteristic	Total patients (*n* = 12)
Age, years	
Mean (SD)	29.8 (19.1)
Range	13–76
Sex	
Male	8 (66.7%)
Female	4 (33.3%)
EASI (total)	
Mean (SD)	31.8 (17.2)
Range	13.7–59.2
EASI (head/neck)	
Mean (SD)	2.7 (1.6)
Range	1.2–6.0
IGA	
Mean (SD)	3.2 (1.1)
Range	3–4
BSA, %	
Mean (SD)	53.8 (27.2)
Range	20–100
NRS	
Mean (SD)	6.9 (2.8)
Range	2–10
POEM	
Mean (SD)	14.6 (6.5)
Range	5–22
Eosinophils, μL	
Mean (SD)	551.7 (343.6)
Range	140–1290
IgE, IU/mL	
Mean (SD)	6692.2 (7959.3)
Range	172–29 305
LDH, IU/L	
Mean (SD)	239.2 (75.7)
Range	171–432
TARC, pg/mL	
Mean (SD)	2770 (3307.7)
Range	674–10 340

Abbreviations: BSA, body surface area, EASI, Eczema Area and Severity Index; IGA, Investigator's Global Assessment; LDH, lactate dehydrogenase; NRS, Numerical Rating Scale; POEM, Patient‐Oriented Eczema Measure; SD, standard deviation; TARC, thymus and activation‐regulated chemokine.

**FIGURE 2:**
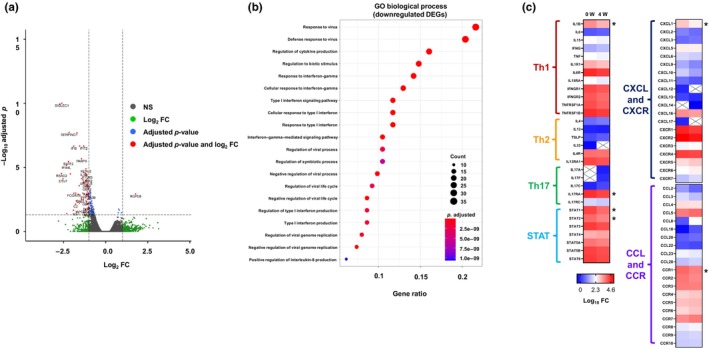
Transcriptome expression analysis of peripheral blood mononuclear cells from three patients with atopic dermatitis (AD) before and after treatment with a 100‐mg dose of abrocitinib. (a) Volcano plot revealed differentially expressed genes (DEGs) at baseline and 4 weeks in patients with AD treated with abrocitinb. (b) Gene Ontology (GO) biological process analysis using downregulated DEGs using QIAGEN's Ingenuity Pathway Analysis. (c) Representative gene expressions of helper T cells Th1, Th2, and Th17 inflammation; signal transducer and activator of transcription (STAT) family; and chemokines. *DEGs. Abbreviations: CCL, CC chemokine ligand; CCR, CC chemokine receptor; CXCL, CXC chemokine ligand; CXCR, CXC chemokine receptor; FC, fold change; NS, not significant.

RNA‐seq analysis identified 182 differentially expressed genes, all of which match a false discovery rate (adjusted *p*‐value) of <0.05. Most of them were downregulated due to abrocitinib treatment (Figure 2a). Gene Ontology group enrichment analysis with the downregulated differentially expressed genes demonstrated defense responses to biotic stimulus, virus, cytokines, and cytokine productions (Figure 2b). Interleukin (IL) 4 expression messenger RNA (mRNA) levels were increased to 32% and IL‐13 was decreased to 13.1% posttreatment. However, the expression of helper T cells Th1, Th2, and Th17 cytokines was not significantly different after abrocitinib treatment, except for IL‐1β. Moreover, abrocitinib treatment suppressed mRNA levels of chemokines, including the chemokine (C‐X‐C motif) ligands CXCL1, CXCL2, CXCL10, and CCL2 (Figure 2c).

## 4 DISCUSSION

In clinical trials, 39.7% to 44.5% and 58.5% to 68.5% of patients who achieved an EASI‐75 response at 12 weeks received monotherapy and combination therapy with topical corticosteroids, respectively.^3^, ^4^, ^5^, ^6^ This retrospective study revealed that EASI‐75 at 12 weeks was lower (41.6%) than in clinical trials, which was almost the same as monotherapy. Some patients did not use the usual dose of corticosteroid ointment from 4 to 12 weeks because the significant improvement in itching discouraged them from continuing topical therapy. It might contribute to the lower EASI‐75 response compared with those obtained by other clinical trials. However, PP‐NRS4, considered to represent a clinically imaginable meaningful improvement,^9^ was achieved in nine patients (75%), which was higher than 47.5% to 52.6% in combination clinical trials.^3^, ^6^ These results indicate that 100 mg of abrocitinib may be useful in the itch‐dominant group with mild to moderate skin rash and a strong perception of itchiness, similar to that in baricitinib.^10^, ^11^ In addition, in another clinical trial, 200 mg of abrocitinib demonstrated a higher therapeutic efficacy for skin rash and pruritus compared with 100 mg of abrocitinib and dupilumab at 12 weeks.^6^ These findings indicate that in patients whose skin rash does not improve adequately with 100 mg of abrocitinib, switching to a double dose would be advisable for both adolescents and adults. However, the increased risk of side effects must also be considered in such cases. The overall frequency of acne in clinical trials was 4.5% with 100 mg of abrocitinib treatment.^12^ The reason for the higher prevalence of acne in our cases may be related to the higher percentage of adolescent to young male patients, and the more stringent requirement in Japan than in other countries to wear masks to prevent COVID‐19 infection.

RNA‐seq analysis revealed evidence of negative immune system regulation against infections, which might support the increased risk of herpes zoster and acne by JAK inhibitors. Recent studies revealed the association between itch or pain and chemokines.^13^, ^14^ Results revealed the decreased expression levels of several chemokines, with CXCL1 especially showing a significant difference. CXCL1 modulates the activity of the transient receptor potential vanilloid 1 (TRPV1), known as the sensory neuron involved in pain and pruritus.^15^ According to these results, abrocitinib might suppress itch via suppressing various types of signaling pathways in addition to blocking JAK–STAT signaling activated by Th2 chemokines in AD.

This study has several limitations, such as a small sample population, short time of observation, and variation in the use of topical agents. The mRNA levels of genes associated with the expression of Th2 inflammation cytokines demonstrated no significant difference at 4 weeks after abrocitinib treatment at a 100‐mg dose. This also might be due to the short‐term observation period in our study. Moreover, RNA‐seq was performed using peripheral blood cells; however, it did not evaluate the changes of barrier dysfunction and local inflammatory response in the dermatitis area in the skin after baricitinib treatment. In conclusion, our results indicate abrocitinib as a highly antipruritic agent that is effective for moderate skin eruptions. Further studies are required to determine the clinical efficacy and safety of long‐term use and drug dosage comparison.

## CONFLICT OF INTEREST

None declared.

## Supporting information


Table S1

Figure S1
