# Supplementary figures and images for: Real‐world effectiveness and safety of abrocitinib in 12 Japanese patients with atopic dermatitis and transcriptome analysis with peripheral blood
Source: J Dermatol. 2024 Mar 3;51(6):849–53. doi: 10.1111/1346-8138.17173 (PMC11483892; doi:10.1111/1346-8138.17173)

Supplementary  
Figure S1

Baseline

4 weeks

12 weeks

#1

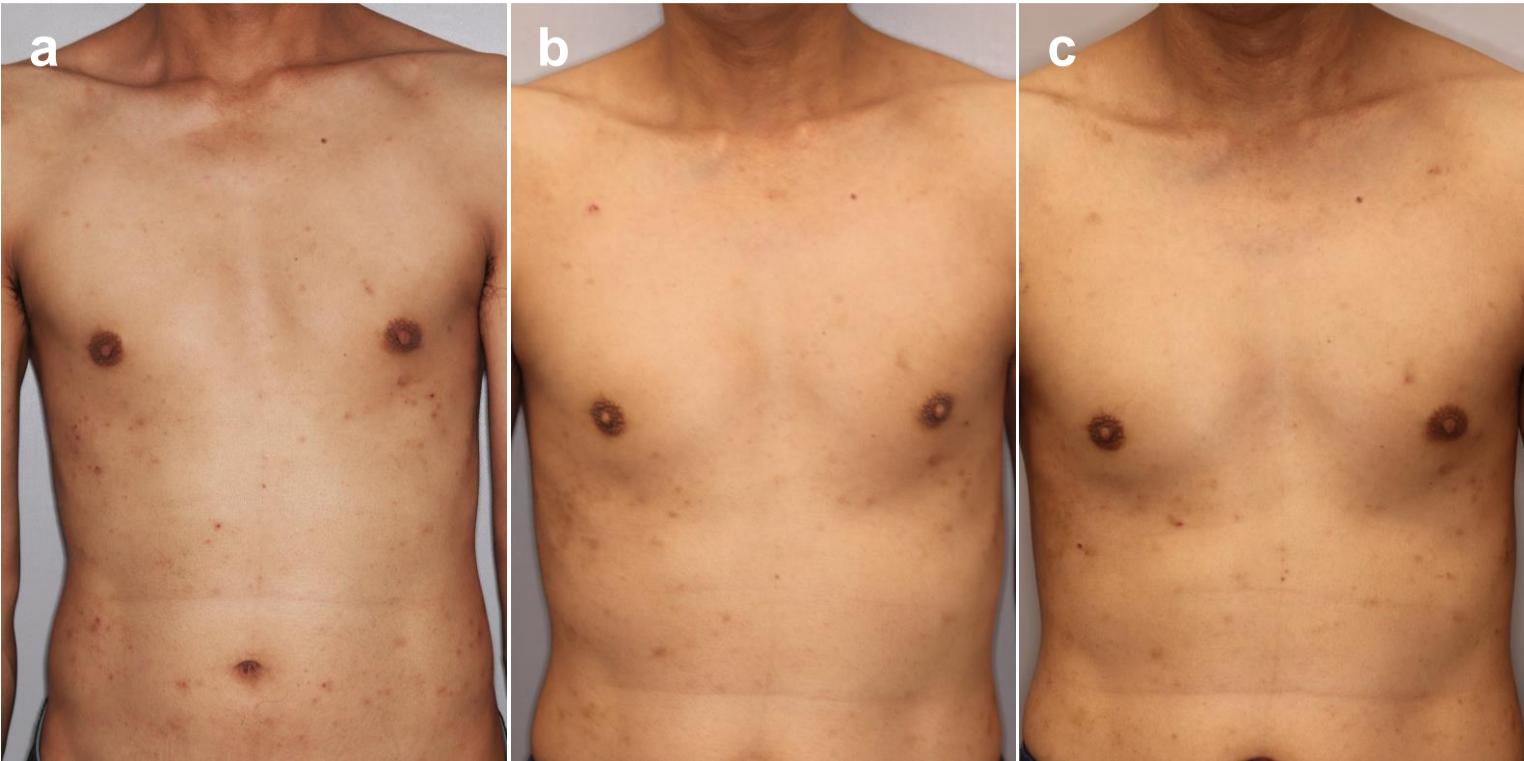

#3

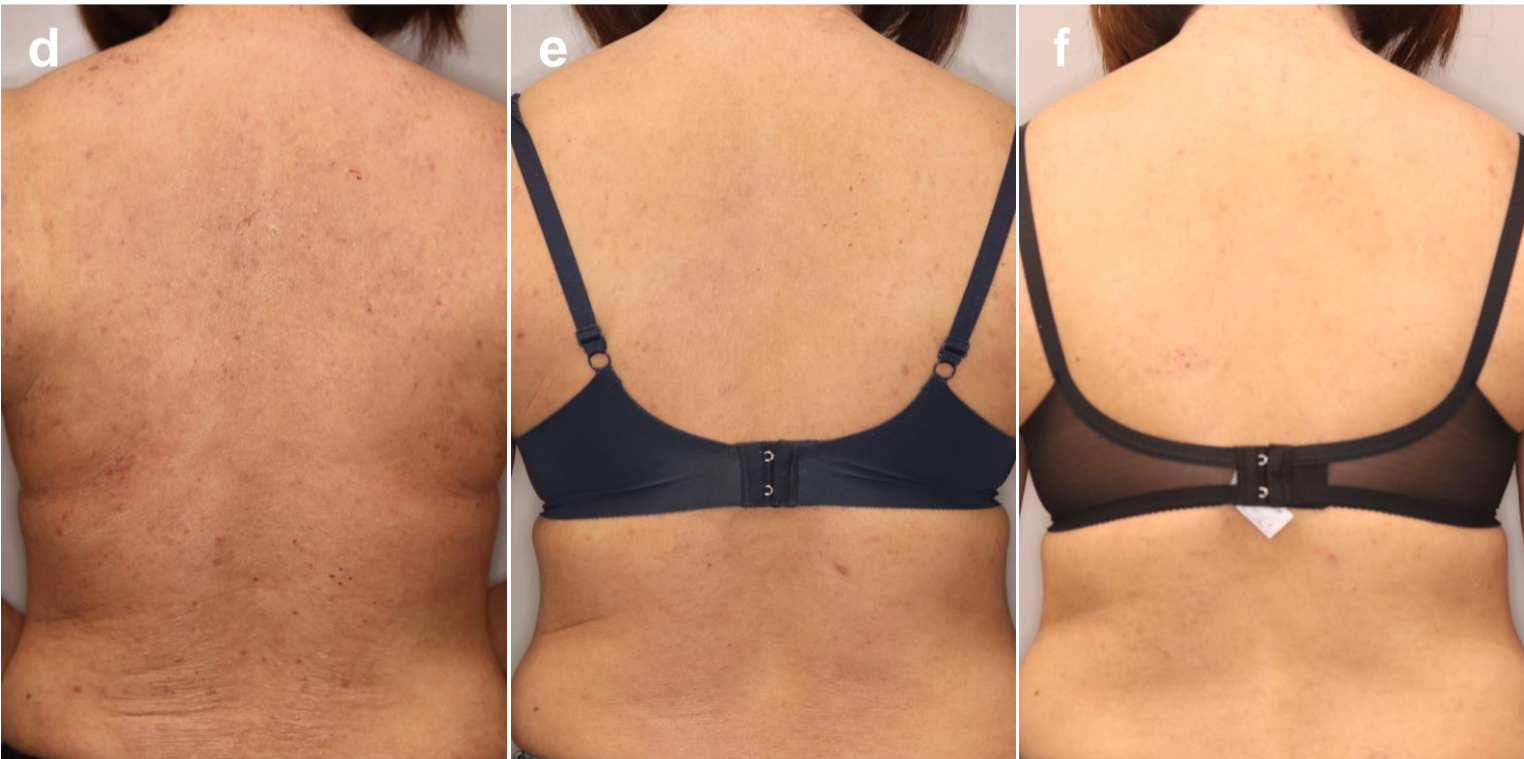

#4

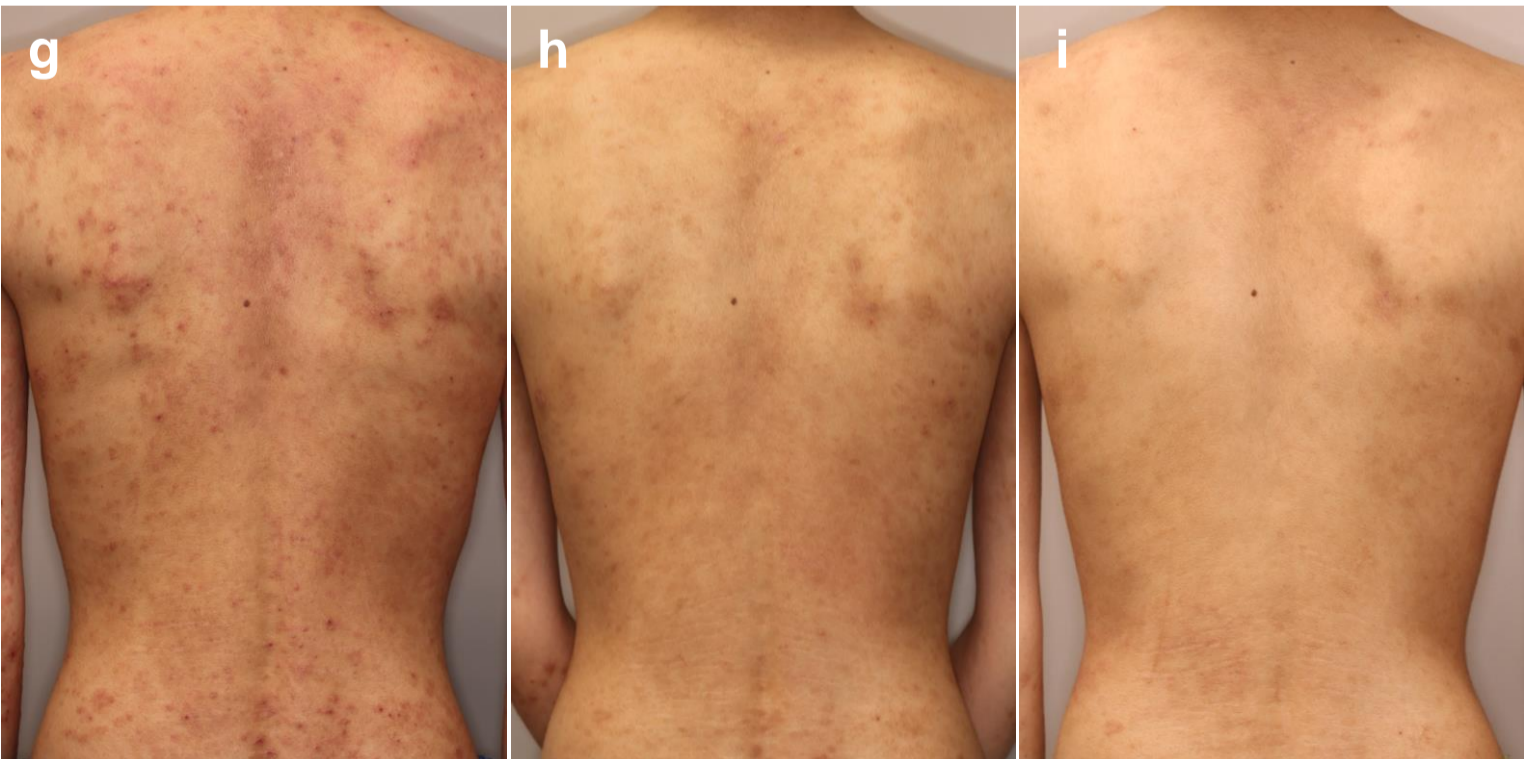

Supplement: Supplementary file 2 — Figure S1 [file JDE-51--s001.pdf]
