# Supplementary material for: Real‐world effectiveness and safety of abrocitinib in 12 Japanese patients with atopic dermatitis and transcriptome analysis with peripheral blood
Source: J Dermatol. 2024 Mar 3;51(6):849–53. doi: 10.1111/1346-8138.17173 (PMC11483892; doi:10.1111/1346-8138.17173)
Supplement: Supplementary file 2 [file JDE-51--s002.docx]

Supplementary Table S1

Details of 12 patients with atopic dermatitis treated with baricitinib

						**Baseline**			
Case	Age	Sex	EASI (total)	EASI (head/neck)	BSA (%)	Eosinophils (/µL)	IgE (IU/mL)	LDH (IU/L)	TARC (pg/mL)
#1	40	M	53.4	4.8	100	140	12280	180	1174
#2	44	M	43.6	6	60	390	5306	216	1021
#3	42	M	59.2	4.4	90	590	29305	432	10340
#4	24	M	40.5	2.1	50	830	5669	302	7228
#5	76	M	39.6	3.6	50	280	3098	302	1765
#6	13	M	29.4	1.4	50	170	172	171	429
#7	18	F	21.6	3.2	50	330	949	183	560
#8	13	M	30.1	2.1	80	1290	8264	177	1082
#9	36	M	17.6	2.4	40	770	5920	247	6528
#10	22	M	46.2	2.7	70	920	1810	240	1137
#11	14	M	18	1.2	40	520	1457	226	674
#12	16	M	13.7	2.4	20	390	3679	195	1302
